# Racial and survival disparities in inflammatory breast cancer (IBC) and non-IBC: a population-based study focused on Native Hawaiians and other Pacific Islanders

**DOI:** 10.3389/fonc.2024.1390080

**Published:** 2024-05-17

**Authors:** Gene T. Yoshikawa, Kyle SY. Miyazaki, Jared D. Acoba, Takeo Fujii

**Affiliations:** ^1^ Department of Medicine, University of Hawai’i Internal Medicine Residency Program, Honolulu, HI, United States; ^2^ John A. Burns School of Medicine, University of Hawai’i at Manoa, Honolulu, HI, United States; ^3^ Cancer Biology Program, Translational and Clinical Research, University of Hawai’i Cancer Center, Honolulu, HI, United States; ^4^ Women’s Malignancies Branch, Center for Cancer Research, National Cancer Institute, National Institutes of Health, Bethesda, MD, United States

**Keywords:** breast cancer, inflammatory breast cancer (IBC), disparities, Native Hawaiian and other Pacific Islander, survival

## Abstract

**Background:**

It is well known that race is an independent predictor of breast cancer mortality and advanced stage at diagnosis. Inflammatory breast cancer (IBC) is the most aggressive type of breast cancer and has distinct clinical and biological features. Previous studies have shown that Blacks have a higher incidence of IBC than Whites. However, the proportion of IBC and the role of race on prognosis in Native Hawaiian and other Pacific Islander (NH/PI) populations with breast cancer are poorly understood. In this study, we aimed to examine the proportion of IBC to non-IBC in NH/PIs and to identify the clinicopathological, biological, and socioeconomic factors associated with the overall survival of NH/PIs compared to other races.

**Methods:**

Utilizing a comprehensive cancer registry from the largest hospital in Hawaii, newly diagnosed primary invasive breast cancer patients diagnosed between 2000 and 2018 were identified. Univariate and multivariate Cox proportional hazards models were used to test the association between race and clinical outcomes. Variables with P-values <0.05 in the univariate analysis and race (variable of interest) were included in a multivariate analysis.

**Results:**

The cohort included 3691 patients, 60 of whom had IBC. NH/PI race had the highest proportion of IBC compared to other races (3.44%) but was not found to be an independent poor prognostic factor in IBC (HR 1.17 [95%CI 0.26-5.22]). Conversely, NH/PI race was associated with worse survival outcomes in patients with non-IBC (HR 1.65 [95%CI, 1.14-2.39]) along with other factors such as lack of insurance, underinsured status, triple-negative breast cancer (TNBC) subtype, age, and advanced clinical stage.

**Conclusions:**

The findings of this study highlight that NH/PIs had higher rates of IBC and inferior survival in non-IBC compared to other races but not in IBC. It is essential to disaggregate NH/PI race from Asians in future population-based research studies. Further research is needed to understand the factors contributing to higher rates of IBC and poor survival outcomes in NH/PIs with non-IBC as well as targeted interventions to improve breast cancer outcomes in this population to ultimately help improve survival rates and reduce health inequities in NH/PIs with breast cancer.

## Introduction

Breast cancer has the highest incidence among all malignancies and is the second leading cause of cancer death among women in the United States (1). According to the American Cancer Society and National Cancer Institute, in 2023, approximately 300,000 new cases and 44,000 deaths occurred in the United States due to breast cancer (1). Health disparities have been a monumental healthcare issue in the United States. For example, given the improvement of screening strategies, the overall incidence of breast cancer has, on average, increased by 0.3% annually from 2004 to 2018, with the increase varying among different races (2). Among Asians and Pacific Islanders, the annual increase was 1.4%, which is significantly higher compared to Whites, whose rate of increase was 0.4% (2). Although the precise cause remains unclear, this could indicate that fewer Asian or Pacific Islanders had recommended screening in the past compared to Whites (2). Additionally, it is well established that race is an independent predictor of mortality and advanced stage at diagnosis (3, 4). These facts suggest the importance of investigating race in cancer research. Unfortunately, not many studies focus on small racial populations such as Native Hawaiians and Pacific Islanders (NH/PI), given that one of the major challenges in studying these populations is the limited number of patients in health databases. Even in the State of Hawaii, only approximately 10% of the residents are of Native Hawaiian and Pacific Islander race (5).

In addition to race, it has been found that lower socioeconomic status (SES) is also associated with increased breast cancer mortality and higher clinical stage at diagnosis after adjusting for other potential confounders (6–9). The National Cancer Institute (NCI) defines SES as a way to classify individuals based on their financial, educational, social, and health-related circumstances (10). Oakes et al. offer a broader definition of SES as “differential access (realized and potential) to desired resources” and acknowledge that it is not as closely related to “occupational class or employment relationships” as other definitions of SES might suggest (11). Their extensive analysis of SES and its measurement in health research confirms a lack of consensus on a universal definition or measurement tool but strongly identifies SES as closely connected with health and disease (11). Prior retrospective reviews of studies investigating cancer care outcomes and SES disparities have attempted to classify SES based on income, educational level, occupation, health insurance status, and/or other measures of deprivation (12).

The interplays between clinicopathologic factors, socioeconomic factors, and race are becoming increasingly recognized (13). However, establishing which element has a more significant influence has yet to be determined (14). Moreover, although there are previous studies investigating this topic, unfortunately, many populational-based studies comprise primarily Black and White racial groups. As a result, little is known about the influence of racial disparities and SES, particularly on the outcomes of NH/PI patients with breast cancer. However, given the trend in the increase in breast cancer rates among Pacific Islanders (15, 16), disaggregating NH/PI patients in epidemiological cancer-based research is paramount (17).

It is also essential to differentiate Inflammatory Breast Cancer (IBC) from non-IBC. IBC is a rare but aggressive form of breast cancer, accounting for only 2-6% of all breast cancers but a disproportionate 7% of breast cancer deaths (18). Additionally, the clinical features of IBC are distinct from those of non-IBC. Approximately 20-30% of patients with IBC present with metastatic disease at diagnosis compared to 6-10% of non-IBC patients (18). Several previous studies demonstrated that the incidence of IBC is highest in Blacks (15–17), which suggests that the incidence of IBC can vary depending on race. However, to the best of our knowledge, it has never been investigated in NH/PI patients, given the rarity of the disease and the limitations of databases, including the small number of NH/PI patients in prior studies. Understanding the epidemiologic characteristics of IBC among NH/PI patients can better provide new insights into the diagnostic and treatment strategies of breast cancer, potentially leading to higher cost-effectiveness and improved health outcomes.

We hypothesize that the proportion of IBC is higher among NH/PI populations compared to other races, and these populations are correlated with poor survival outcomes. In this study, our primary objective was to identify and compare the proportion of IBC and non-IBC patients among distinct races (including PI and NH). The secondary objective was to identify the clinicopathological, biological, and socioeconomic factors associated with overall survival in patients with IBC and non-IBC separately.

## Materials and methods

### Design and sample

This single-center retrospective chart review observational study was approved, and informed consent was waived due to the study’s retrospective nature by the Queen’s Medical Center’s Institutional Review Board (protocol number: RA-2019-027). We used Queen’s Medical Center Tumor Registry to identify patients with newly diagnosed primary invasive breast cancer who were diagnosed between January 1, 2000, and December 31, 2018, which is the time frame that the data set covers. The patients diagnosed with other types of co-existing cancers were excluded. Since we were interested in the effects of factors related to breast cancer on overall survival, we decided to exclude patients with co-existing cancers to eliminate the possibility of death from other types of aggressive cancers. Although OS as a definition includes any cause of death, unfortunately breast cancer-specific death would not have been reliably available from our data set, and OS was therefore used as an alternative.

### Data extraction

The data was extracted from the Queen’s Medical Center Tumor Registry data base by the data manager and the patients with invasive breast cancer without co-existing cancers were identified. From the tumor registry, we extracted age at diagnosis, race (White, Asian, Native Hawaiian or Pacific Islander, or Others based on the U.S. Office of Management and Budget (OMB) standard), primary insurance (private insurance, MEDICARE, MEDICAID, others, or no insurance), histology (ductal, lobular, mixed, or others), the proportion of IBC vs. non-IBC, clinical stage, estrogen receptor (ER) and/or progesterone receptor (PR) by immunohistochemical staining, and human epidermal growth factor receptor 2 (HER2) status and date of death or last follow-up. ER and PR positivity were defined based on American Society of Clinical Oncology (ASCO)/College of American Pathologists (CAP) guidelines (19). HER2 positivity was defined as a HER2/CEP17 fluorescence *in situ* hybridization (FISH) ratio of ≥2.0 and/or an immunohistochemical (IHC) staining score of 3+. Of note, Black race is uncommon in Hawaii and constituted a very small proportion of our study population; consequently, it was grouped under the broader category of “Others” for the purposes of our analysis although we are aware that Black race is an important race in IBC.

Our primary objective was to identify and compare the proportion of IBC and non-IBC patients among distinct races (including PI and NH), which is defined as the ratio of the number of patients with IBC to that of those with all breast cancers. The secondary objective was to identify the clinicopathological, biological, and socioeconomic factors associated with overall survival in patients with IBC and non-IBC separately. The collected variables are listed above.

### Statistical analysis

To summarize the baseline characteristics table, standard descriptive statistics and frequency tabulation were used. The chi-square and Fisher’s exact tests were used to assess the association between two categorical variables depending on expected values. The Kruskal-Wallis test was used to compare the distribution of continuous variables between different groups.

For our primary objective analysis, the proportion of patients with IBC was calculated by the ratio of the number of patients with IBC to those with all breast cancers among each race. To compare the proportion in Whites to that in NH/PI, the Fisher’s exact test was used. For our secondary objective analysis, univariate Cox proportional hazards model was used to investigate the association between each variable and overall survival (OS) for variable selection. OS was defined as the time from diagnosis to death. Age, race based on OMB standard, primary insurance, histology, clinical stage, and subtype which was categorized as “HR-positive/HER2-negative” defined as positivity for ER and/or PR and negativity for HER2, “HER-2 positive” defined as negativity for both ER and PR and positivity for HER2, and “TNBC” defined as negativity for all ER, PR, and HER2,were assessed as independent variables in univariate Cox proportional hazards model and only the variables with P-value<0.05 and race, which is the variable of our interest, were included in the multivariable Cox proportional hazards model. Patients who were alive at the date of the last follow-up were censored.

“Unknown” group in the Race category, “Unknown” group in the Primary Insurance category, “Others” and “Unknown” groups in the Histology category, “Unknown” group in the Subtype category, and “Unknown” group in the Clinical Stage category were not included in the analyses. All tests were two-sided. P-values <0.05 were considered statistically significant. STATA, version 14 (STATA Corp, College Station, TX), was used for all analyses.

## Results

### Patient characteristics

A total of 3691 patients were identified. Of those 3691 patients, 60 (1.5%) had IBC (**Table 1**). Twenty-six out of 60 patients with IBC (43.3%) were NH/PI. The overall proportion of NH/PI race was less in patients with non-IBC (730 of 3631 patients with non-IBC; 20.1%). Thirty-six of 60 patients with IBC (60%) had stage III disease. Of note, there is no stage I or II disease in IBC. The subtype was unknown for approximately 35% of patients with non-IBC because this information was recorded in a different non-EMR Pathology Department system, which could not be collected. Thirty of 60 patients with IBC (50%) and 2131 of 3631 patients with non-IBC (58.7%) had private insurance.

**Table 1:** Baseline patient characteristics.

	IBC (N=60) n (%)	Non-IBC (N=3631) n (%)	P-value
**Age (Median, range)**	59 (29-72)	59 (22-96)	0.11
**Race**			<0.001
** White**	12 (20)	712 (19.6)	
** Asian**	20 (33.3)	2071 (57)	
** Native Hawaiian or Pacific Islander**	26 (43.3)	730 (20.1)	
** Others**	2 (3.3)	112 (3.1)	
** Unknown**	0 (0)	6 (0.2)	
**Primary Insurance**			<0.001
** Private insurance**	30 (50)	2131 (58.7)	
** Medicare**	12 (20)	1053 (29)	
** Medicaid**	12 (20)	245 (6.7)	
** Others**	3 (5)	168 (4.6)	
** No insurance**	3 (5)	21 (0.6)	
** Unknown**	0 (0)	13 (0.4)	
**Histology**			0.3
** Ductal**	50 (83.3)	3183 (87.7)	
** Lobular**	1 (1.7)	233 (6.4)	
** Mixed**	0 (0)	45 (1.2)	
** Others**	0 (0)	146 (4)	
** Unknown**	9 (15)	24 (0.7)	
Clinical Stage*			1.0
** Stage I**	N/A	2103 (57.9)	
** Stage II**	N/A	1057 (29.1)	
** Stage III**	36 (60)	224 (6.2)	
** Stage IV**	20 (33.3)	131 (3.6)	
** Unknown**	4 (6.7)	116 (3.2)	
*Only Stages III and IV were used in the IBC analysis since there is no Stage I or II in IBC			
**Subtype**			<0.001
** HR-positive/HER2-negative**	23 (38.3)	1790 (49.3)	
** HER2-positive**	22 (36.7)	316 (8.7)	
** TNBC**	6 (10)	225 (6.2)	
** Unknown**	9 (15)	1300 (35.8)	

HR, hormone receptor; HER2, human epidermal growth factor receptor 2; TNBC, triple negative breast cancer.

*Only Stages III and IV were used in the IBC analysis since there is no Stage I or II in IBC.

### Racial difference in the proportion of patients with IBC to those with non-IBC

The proportion of those with IBC was highest in the NH/PI race, with 26 of 756 NH/PI having IBC (3.44%). The proportion of Whites with IBC was 1.66% (12 of 712 patients). Compared to the proportion of Whites, the NH/PI race had a significantly high proportion of IBC (P=0.003) (**Table 2**).

**Table 2:** Proportion of IBC by Race.

Race	IBC (N=60)	Non-IBC (N=3631)	% of IBC among all BC cases
** White**	12	712	1.66
** Asian**	20	2071	0.96
** Native Hawaiian or Pacific Islander**	26	730	3.44
** Others**	2	112	1.75

### Overall survival in patients with IBC and those with non-IBC

The median follow-up of the OS was 63 months. Among the patients with IBC, in a univariate Cox proportional hazard model, no insurance (HR, 5.38 [95%CI, 1.47-19.7]; P=0.01), MEDICAID (HR, 3.83 [95%CI, 1.58-9.28]; P<0.01), TNBC subtype (HR, 5.43 [95%CI, 1.66-17.73]; P<0.001), and clinical stage IV disease (HR, 10.58 [95%CI, 4.06-27.59]; P<0.001) were associated with shorter OS, but NH/PI race (HR, 2.46 [95%CI, 0.81-7.45]; P=0.11) was not. In a multivariable Cox proportional hazard model adjusting for the variables with P-values < 0.05 in the univariate analysis and race, MEDICAID insurance (HR, 4.45 [95%CI, 1.1-18.03]; P=0.04), TNBC subtype (HR, 4.46 [95%CI, 1.08-19.5]; P=0.04), and clinical stage IV disease (HR, 10.1 [95%CI, 2.35-43.4]; P<0.001) remained significant. NH/PI race remained a non-significant factor associated with shorter OS (HR, 1.17 [95%CI, 0.26-5.223]; P=0.84) (**Table 3**). Kaplan-Meier survival curve for OS is shown in **Supplementary Figure 1A**.

**Table 3:** Univariate and multivariate analysis for OS among patients with IBC. (N=60).

	Univariate		Multivariate	
	HR (95% CI)	P-value	HR (95% CI)	P-value
**Age**	1.0 (0.97-1.03)	0.94		
Race				
** White**	Ref		Ref	
** Asian**	2.1 (0.66-6.62)	0.21	0.6 (0.11-3.09)	0.54
** Native Hawaiian or Pacific Islander**	2.46 (0.81-7.45)	0.11	1.17 (0.26-5.22)	0.84
** Others**	1.34 (0.15-12.01)	0.8	27.85 (1.81-427.97)	0.02
Primary Insurance				
** Private insurance**	Ref		Ref	
** MEDICARE**	1.25 (0.47-3.33)	0.66	0.59 (0.12-2.89)	0.51
** MEDICAID**	3.83 (1.58-9.28)	<0.01	4.45 (1.1-18.03)	0.04
** Others**	0.64 (0.08-4.98)	0.67	0.64 (0.07-5.56)	0.68
** No insurance**	5.38 (1.47-19.7)	0.01	4.08 (0.64-26.2)	0.14
Histology				
** Ductal**	Ref			
** Lobular**	1.29 (0.17-9.64)	0.81		
Subtype				
** HR-positive/HER2-negative**	Ref		Ref	
** HER2-positive**	1.16 (0.48-2.78)	0.75	0.65 (0.22-1.9)	0.43
** TNBC**	5.43 (1.66-17.73)	<0.001	4.6 (1.08-19.5)	0.04
Clinical Stage				
** Stage III**	Ref		Ref	
** Stage IV**	10.58 (4.06-27.59)	<0.001	10.1 (2.35-43.4)	<.001

Similarly to the findings in patients with IBC, among those with non-IBC, in the univariate Cox proportional hazard model, no insurance (HR, 5.13 [95%CI, 2.63-9.99]; P<0.001), MEDICAID (HR, 3.1 [95%CI, 2.29-4.17]; P<0.01), TNBC subtype (HR, 2.38 [95%CI, 1.78-3.19]; P<0.01), and advanced clinical stage were associated with shorter OS. Additionally, NH/PI race (HR, 1.43 [95%CI, 1.13-1.81]; P<0.001) and age (HR, 1.05 [95%CI, 1.04-1.06]; P<0.01) were also associated with shorter OS (**Table 4**). In a multivariable Cox proportional hazard model adjusting for the variables with P-values < 0.05, no insurance (HR, 3.34 [95%CI, 0.94-2.81]; P=0.04), MEDICAID insurance (HR, 1.79 [95%CI, 1.15-2.77]; P=0.01), TNBC subtype (HR, 1.76 [95%CI, 1.3-2.41]; P<0.01), age (HR, 1.05 [95%CI, 1.04-1.07]; P<0.01), advanced clinical stage (HR, 10.1 [95%CI, 2.35-43.4]; P<0.001), and NH/PI race (HR, 1.65 [95%CI, 1.14-2.39]; P=0.007) remained significant (**Table 4**). Kaplan-Meier survival curve for OS is shown in **Supplementary Figure 1B**.

**Table 4:** Univariate and multivariate analysis for OS among patients with non-IBC. (N=3631).

	Univariate		Multivariate	
	HR (95% CI)	P-value	HR (95% CI)	P-value
**Age**	1.05 (1.04-1.06)	<0.01	1.05 (1.04-1.07)	<0.01
Race				
** White**	Ref		Ref	
** Asian**	0.87 (0.7-1.07)	0.19	0.83 (0.6-1.14)	0.244
** Native Hawaiian or Pacific islander**	1.43 (1.13-1.81)	<0.001	1.65 (1.14-2.39)	0.007
** Others**	1.34 (0.86-20.9)	0.86	1.34 (0.63-2.84)	0.45
Primary insurance				
** HMSA**	Ref		Ref	
** MEDICARE**	3.4 (2.84-4.08)	<0.01	1.35 (0.94-1.95)	0.1
** MEDICAID**	3.1 (2.29-4.17)	<0.01	1.79 (1.15-2.77)	0.01
** Others**	1.82 (1.3-2.55)	<0.01	1.63 (0.94-2.81)	0.08
** No insurance**	5.13 (2.63-9.99)	<0.01	3.34 (1.03-10.8)	0.04
Histology				
** Ductal**	Ref			
** Lobular**	1.0 (0.7-1.42)	0.98		
** Mixed**	1.21 (0.66-2.2)	0.54		
** Others**	1.07 (0.74-1.54)	0.72		
Subtype				
** HR-positive/HER2-negative**	Ref		Ref	
** HER2-positive**	1.48 (1.05-2.09)	0.02	1.01 (0.7-1.46)	0.94
** TNBC**	2.38 (1.78-3.19)	<0.01	1.76 (1.3-2.41)	<0.01
Clinical Stage				
** Stage I**	Ref		Ref	
** Stage II**	1.95 (1.59-2.38)	<0.01	2.08 (1.53-2.82)	<0.01
** Stage IIII**	4.38 (3.36-5.72)	<0.01	4.63 (3.14-6.83)	<0.01
** Stage IV**	22.77 (17.68-29.3)	<0.01	26.59 (18.3-38.64)	<0.01

## Discussion

To our knowledge, this is the first study to investigate the association between patient, race, disease characteristics, socioeconomic status, and OS in a unique population focused on Native Hawaiian or other Pacific Islanders (NH/PI) with IBC and non-IBC separately. Although even amongst the NH/PI group, there are geographic and regionalization considerations, we referred to the U.S. Office of Management and Budget (OMB) standard. In accordance with our hypothesis, in our study, we found that NH/PI race had the highest proportion of IBC (3.44%) amongst other races and was statistically significant when compared to the ratio of Whites (P=0.003). Among patients with non-IBC, weak or no insurance was associated with poor OS, which is consistent with previous studies on the influence of SES on survival outcomes (6–8). NH/PI race also remained a significant factor associated with OS. However, among patients with IBC, only TNBC subtype and Stage IV disease were significantly associated with OS, whereas NH/PI was not associated with OS.

Despite NH/PI race having a significantly higher proportion of IBC than other races, NH/PI race was not found to be an independent poor prognostic factor amongst those with IBC. Although the reason for this lack of difference in survival in IBC is currently unclear, we suspect the aggressive nature of IBC is likely one of the highly contributory factors. Small sample size might be another reason. Previous studies have consistently shown that for those with IBC, Black race is associated with poor prognosis (7, 20, 21). However, other races have not reliably been shown to be independent poor prognostic factors in those with IBC (21). One possible reason for this finding is that this could indicate unique disparities (e.g., biological variations) amongst Blacks with IBC that do not necessarily affect other races (including NH/PI race) regardless of the prevalence of the disease. However, given the rarity of IBC, small sample size should again be considered when interpreting these results. In our study, MEDICAID insurance was also associated with worse survival amongst those with IBC, although no insurance was not a significant factor. This is likely due to small sample size (only three patients with IBC had no insurance) because patients without insurance coverage face similar challenges to patients with MEDICAID insurance, such as access to care and lower income levels.

Contrary to that stated above, NH/PI race was found to be associated with worse OS in those with non-IBC in our study. MEDICAID or uninsured, TNBC sub-type, and advanced clinical stage were also found to be poor prognostic factors. Although the reason why NH/PI race is an adverse prognostic factor is unclear, it is likely multifactorial, influenced by both biological and socioeconomic causes. Uninsured and underinsured status are likely substantial contributory factors, although NH/PIs have also been found to have poor health outcomes despite being insured (22). It has been well established that low socioeconomic status (SES) is associated with worse outcomes in patients with cancer (6–8, 23). Among men and women, five-year survival for all cancers combined is ten percentage points lower than those of higher SES (23). Unfortunately, NH/PI populations have been associated with lower SES, with approximately 15% of NH/PIs living in poverty compared to 11% of Asians or 13% of Americans overall (24). Low SES can result in lower quality of life (resulting in higher health risks), fragmentation of care, complications with health insurance, lower education, health literacy, and less access to care (25). For example, Sentell et al. found that low health literacy was a significant predictor of poorer health outcomes in adults of NH/PI race (26). Additionally, Taparra et al. found that within a total cohort of almost 600,000 women with stage 0-II breast cancer, NH/PI women had worse survival when compared with non-Hispanic White women (27). NH/PI women had consistently longer times between surgery and radiation therapy. Thus, delays in care were suggested to be a significant contributory factor to the finding of increased mortality in this population (27). These delays in care are suspected to primarily result from poor access to care in NH/PI populations due to financial or geographical hardships. Not only does this apply to local NH/PI patients in the United States but also to the majority of Pacific Islanders originating from Pacific Island Countries where medical care is limited. This requires these patients to travel long distances and at a significant cost to seek appropriate medical treatment, especially when specialty or hospital-based care is needed (28). Subsequently, this leads to even further fragmentation of care, given that many patients have family and friends in their home countries, requiring frequent travel back, which can ultimately interrupt treatment plans as well. If efforts are made to address these socioeconomic disparities, such as improved access to health care, financial assistance programs, or culturally appropriate support services, perhaps patients could be diagnosed at earlier stages of the disease, ultimately improving health outcomes and cost-effectiveness (29).

In addition to socioeconomic causes, biological etiologies are also a consideration for the finding of non-IBC as an independent poor prognostic factor amongst NH/PIs in our study. Previous research has demonstrated higher incidence rates of both hormone receptor (HR) and human epidermal growth factor receptor 2 (HER2) expression in Native Hawaiians (30–32). HR-positive breast cancers generally tend to have a better prognosis than HR-negative breast cancers (33). However, HER2 overexpression in invasive breast cancers is associated with higher rates of disease recurrence, brain metastasis, and mortality (34). HER2-positive breast cancers have also been found to have the second poorest prognosis amongst all breast cancer subtypes (35). Since the advent of HER2-targeted therapies such as trastuzumab or pertuzumab, there has been a paradigm shift amongst patients with HER2-positive breast cancer, resulting in decreased mortality rates. Still, if NH/PI patients cannot receive these therapies due to poor access to care or other socioeconomic barriers, as discussed above, then HER2 overexpression could be a biological explanation for this finding. However, further investigation is necessary to determine the impact of receptor expression on mortality in NH/PIs with invasive breast carcinoma.

Gaining a deeper understanding of how certain breast cancer therapies will impact NH/PIs can facilitate the prediction of medication toxicity within this demographic as well. For instance, in a meta-analysis by Hirko et al., which investigated the toxicity profiles of patients treated with cyclin-dependent kinase (CDK) 4/6 inhibitors in the MONALEESA-2 (ribociclib + letrozole vs. placebo + letrozole) (36) and PALOMA-2 (palbociclib + letrozole vs. placebo + letrozole) trials (37), it was found that Asians had a higher incidence of neutropenia compared to non-Asians (90.9% vs. 75.1%, *p <* 0.001) (38). Conducting similar analyses and research specifically for NH/PI patients could potentially influence treatment decisions or recommendations, as well as intensify monitoring parameters for these patients. Furthermore, in an era of personalized medicine, understanding the genetic profiles of NH/PI populations could provide insight into prognosis, familial risk of disease occurrence, and treatment options. For instance, studies have revealed that Ashkenazi Jewish individuals are at higher risk of harboring detrimental BRCA1 or BRCA2 mutation, whereas Black women exhibit BRCA1 variants unique to their racial group (39). Given that inheritance of a BRCA1 or BRCA2 mutation correlates with earlier onset of disease, aggressive tumor behavior, and heightened recurrence risk, targeted therapies such as oral poly(ADP-ribose) polymerase (PARP) inhibitors offer potential additional treatments for these patients (40). To our knowledge, there are no known dedicated studies focusing on assessing the prevalence of germline or somatic mutations in NH/PI breast cancer patients.

In our study, several limitations should be considered when interpreting our results. First, this was a retrospective chart review study. Although we controlled for race, insurance status, histology, and clinical stage, given the nature of the study, there are potential unknown confounding factors as well as other variables that were unable to be collected (e.g., socio-economic status, distance to health care facilities, family support, and income), which could have affected our results. Socioeconomic status (SES) is a complex construct within health research and may be classified through ecologic measures of social deprivation as well as measures of income, educational level, occupation, and/or health insurance status (12). While our study is limited by these potential compounding factors of SES, we did attempt to address this by controlling for insurance status, which has previously been utilized as a surrogate or proxy measure of SES in assessing survival outcomes in cancer (41–45) (41–45).Although the use of health insurance status is an acceptable surrogate for SES, potential confounding may occur (46). Second, we were unable to account for how many of the patients included in the study were actual residents of Hawaii vs. non-residents in the surrounding archipelagoes who had sought to receive treatment in a more developed health system. Third, although the sample size of the patients with non-IBC was favorable, we could only include sixty patients with IBC in our analysis. Unfortunately, this small sample size reduces the power of the study to identify slight differences and variations. However, as previously discussed, IBC is a rare form of breast cancer and can be challenging to observe, especially in smaller populations such as in Hawaii. Fourth, not all data was present for all patients included in the analysis. Notably, as mentioned above, the subtype of breast cancer was unknown for approximately 35% of patients with non-IBC due to the inability to collect the data from the non-EMR Pathology Department system. Fifth, it is important to note that this study was conducted at a single center. Unlike multi-center studies, single-center studies often involve smaller sample sizes and may lack the generalizability and external validity needed to apply findings to broader populations (47). In our study, we obtained our data from the Queen’s Medical Center (QMC) Tumor Registry. The Queen’s Medical Center is the largest Hospital in Hawaii and manages the most Oncology patients in the state (48). Although we acknowledge the limitations inherent in single-center studies, given that our institution manages a significant portion of the Oncology patient population (including breast cancer patients) in all of Hawaii, we are optimistic that our findings would be representative of similar trends found at other institutions. Sixth, the patient population included in our study was diagnosed with breast cancer between 2000 and 2018. The standard of care for those patients could have been different from the current standard of care, which could potentially affect survival outcomes. Lastly, there might be errors in the data set because the information was manually recorded in the registry database.

In conclusion, our study demonstrated that NH/PI race had a significantly high proportion of IBC when compared to other races. NH/PI race was an adverse prognostic factor associated with worse OS in those with non-IBC but not in those with IBC. In patients with non-IBC, lack of insurance or underinsured status were also associated with shorter OS. Additional research needs to be conducted to further understand the unique determinants and disparities contributing to poor survival outcomes in NH/PI populations, particularly with non-IBC. Unfortunately, there are not many large, multi-center studies that focus on NH/PI populations, and the research that does include NH/PIs typically aggregates this population with Asians. However, as this study has demonstrated, disaggregating NH/PI race from Asians in population-based research is essential. By further identifying these factors, targeted interventions can be implemented to ultimately help improve survival rates and reduce health inequities in NH/PIs with breast cancer.

## Data availability statement

The original contributions presented in the study are included in the article/**Supplementary Material**. Further inquiries can be directed to the corresponding author.

## Ethics statement

The studies involving humans were approved by the Declaration of Helsinki. This retrospective chart review study was approved by the Queen’s Medical Center’s Institutional Review Board (RA-2019-027). Informed consent was waived due to the retrospective nature of the study. The studies were conducted in accordance with the local legislation and institutional requirements. Written informed consent for participation was not required from the participants or the participants’ legal guardians/next of kin in accordance with the national legislation and institutional requirements.

## Author contributions

GY: Data curation, Investigation, Methodology, Resources, Writing – original draft. KM: Data curation, Investigation, Methodology, Resources, Writing – review & editing. JA: Data curation, Formal Analysis, Investigation, Methodology, Resources, Writing – review & editing. TF: Conceptualization, Data curation, Formal Analysis, Funding acquisition, Investigation, Methodology, Resources, Software, Supervision, Writing – review & editing.

## Supplementary material

Supplementary Figure 1 Overall survival (OS) analysis. (a) IBC patients (N=56) (b) non-IBC patients (N=3625).
