# Supplementary figures and images for: Racial and survival disparities in inflammatory breast cancer (IBC) and non-IBC: a population-based study focused on Native Hawaiians and other Pacific Islanders
Source: Front Oncol. 2024 May 17;14:1390080. doi: 10.3389/fonc.2024.1390080 (PMC11140018; doi:10.3389/fonc.2024.1390080)

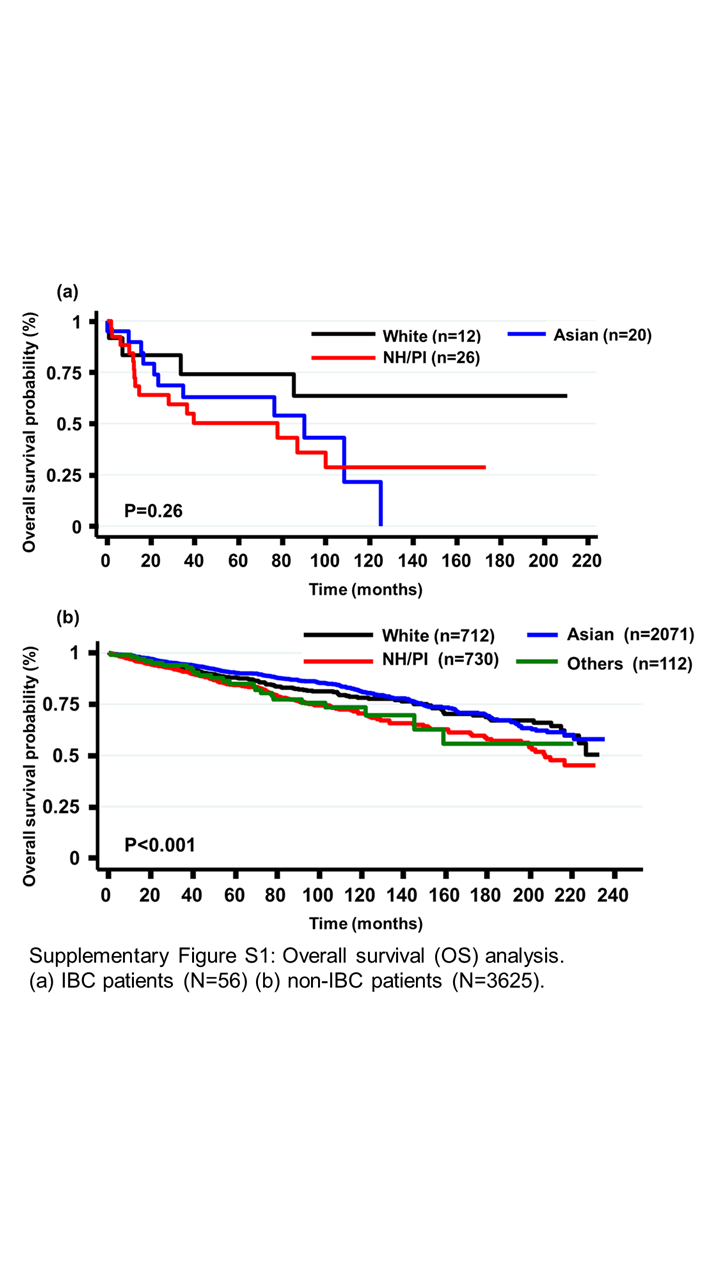

Supplement: Supplementary Figure 1 — Overall survival (OS) analysis. (a) IBC patients (N=56) (b) non-IBC patients (N=3625). [file Image_1.tif]
